# Extensive dynamics of *Plasmodium falciparum *densities, stages and genotyping profiles

**DOI:** 10.1186/1475-2875-7-241

**Published:** 2008-11-21

**Authors:** Anna Färnert, Marianne Lebbad, Lea Faraja, Ingegerd Rooth

**Affiliations:** 1 Unit of Infectious Diseases, Department of Medicine, Karolinska Institutet, Karolinska University Hospital, S-17176 Stockholm, Sweden; 2 Department of Parasitology, Mycology and Environmental Microbiology, Swedish Institute for Infectious Disease Control, Solna, Sweden; 3 Nyamisati Malaria Research, National Institute of Medical Research, Dar es Salaam, Tanzania

## Abstract

**Background:**

Individuals living in areas of high malaria transmission often have different *Plasmodium falciparum *clones detected in the peripheral blood over time. The aim of this study was to assess the dynamics of asymptomatic *P. falciparum *infections in a few hours intervals.

**Methods:**

Capillary blood samples were collected 6-hourly during five days from asymptomatic children in a highly endemic area in Tanzania. Parasite densities and maturation stages were investigated by light microscopy. Types and number of clones were analysed by PCR based genotyping of the polymorphic merozoite surface proteins 1 and 2 genes. Results: Parasite densities and maturation stages fluctuated 48-hourly with a gradual shift into more mature forms. Various genotyping patterns were observed in repeated samples over five days with only few samples with identical profiles. Up to six alleles differed in samples collected six hours apart in the same individual.

**Conclusion:**

This detailed assessment highlights the extensive within-host dynamics of *P. falciparum *populations and the limitations of single blood samples to determine parasite densities, stages and genotyping profiles in a malaria infected individual.

## Background

Longitudinal studies with repeated genotyping of *Plasmodium falciparum *infections in partially immune individuals in areas of high malaria transmission have revealed that different parasites are often detected in their peripheral blood over time (days-months) [1-4]. These changes are a result of new inoculations, immune clearance and intake of anti-malarial drugs. Moreover, within-host dynamics result in that all parasite types infecting an individual may not be present in the peripheral blood at the time of blood sampling [2-4].

Characterization of *P. falciparum *populations is widely performed by PCR genotyping based on the polymorphism of the merozoite surface proteins 1 and 2 genes (*msp1 *and *msp2*). The method is used in molecular epidemiology studies of malaria to define the types and number of genotypes, i.e. clones, in relation to factors such as transmission intensity, age and host immunity. Also in anti-malarial drug trials, genotyping is used to determine whether recurrent parasitaemias are due to new infections or to recrudescence of the initial parasites suggesting treatment failure [5,6].

A wide number of studies have been published with genotyping of *msp1 *and *msp2 *using different protocols. Most reports are based on single time assessments. However, analysis of daily samples from children with asymptomatic *P. falciparum *infections detected different genotypes on consecutive days suggesting interesting within-host dynamics of parasite populations [3,4]. The dynamics need to be considered when interpreting results from studies of *P. falciparum *genotyping. The informative value of single blood samples for determination of the parasite population in an individual at a given time, as well as how quickly can *msp1 *and *msp2 *genotyping patterns change in an individual, is yet to be established. Here, the dynamics of *P. falciparum *infections was assessed in blood samples collected 6-hourly over five days in three children in an area of high transmission. In addition to genotyping of *msp1 *and *msp2*, total parasite densities and maturation stages were established by microscopy. The genotyping profiles, parasite densities and proportions of different maturation stages in samples collected only a few hours apart reflect extensive dynamics of *P. falciparum *in asymptomatic individuals, which need to be considered in various aspects of malaria research.

## Methods

The study was part of a longitudinally malaria project in Nyamisati, a fishing village in the Rufiji River Delta, coastal Tanzania [7]. Malaria was highly endemic in the area, with perennial transmission and peaks around the long and short rain periods. Ethical approval for the project was received from National Institute for Medical Research in Tanzania. Informed consent was obtained from the children and their parents throughout the study. Finger-prick blood samples were collected every six hours (8 am, 2 pm, 8 pm) during a five day period from three asymptomatic children (7, 9 and 11 years old). A nightly sample at 2 am was collected once on day 3. At each sampling occasion, a thick and a thin blood film were prepared for microscopy and 50 microliter of blood was drawn into a capillary tube with citrate as anticoagulant. The blood films were stained in Giemsa and analysed by light microscopy at 1000 × magnification; in thin films against the number of erythrocytes, and in thick films in 200 microscopic fields, assuming a blood volume of 0.2 microliter Morphological staging of the asexual parasites was performed in the thin film according to Silamut & White [8]. The slides were coded and analysed twice by two microscopists blinded for the chronological order of the slides.

Genotyping of *P. falciparum *was performed by nested PCR of the *msp1 *(block2) and *msp2 *(block 3) genes [9]. These unlinked single copy genes are highly polymorphic with varying number of tandem repeats conferring size polymorphism. The three allelic types of *msp1 *(K1, MAD20 and RO33 types) and the two types of *msp2 *(FC27 and IC types) were targeted with specific primers in separate nested reactions. The PCR analyses were performed on genomic DNA purified by phenol/chloroform extraction. The first steps of purification, including red cell lysis with saponin, centrifugation and resuspension of the pellet in lysis buffer (40 mM Tris, 80 mM EDTA, 2% SDS), were performed on the days of collection and the partly processed samples were then frozen at -20°C until further DNA extraction. The amount of DNA analysed in each PCR reaction corresponded to 5 microliter of whole blood. The PCR products were size separated by gel electrophoresis on high resolution Metaphor^® ^Agarose (FMC Bioproducts, Rockland, USA) and visualized by UV-transillumination after ethidium bromide staining.

## Results

**Figure 1:**
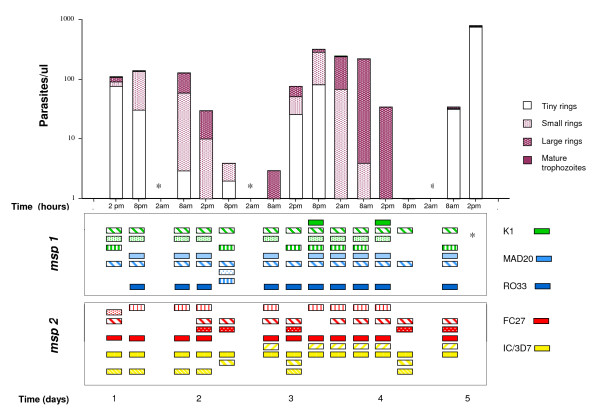
***Plasmodium falciparum *population dynamics in an asymptomatic 9 year old child in 6-hourly samples over 5 days**. Parasite densities and morphologic stages determined by microscopy of Giemsa-stained thin and thick smears. Parasite populations were characterized by PCR based genotyping of *msp1 *and *msp2*. PCR products of the same base pair length within the respective allelic types were defined as the same alleles and schematically illustrated as boxes with the same patterns and colours. The respective allelic types of *msp1 *and *msp2 *are illustrated by different colours. *not done/missing sample

In two children, malaria parasites were not detected by microscopy or PCR in any sample during the five day period. The third child (nine years old) had *P. falciparum *parasites in 13 samples by microscopy and in all consecutive samples by PCR (Figure 1). The total parasite densities fluctuated according to a sine wave curve and ranged between 0 and 790 asexual parasites/microliter. Only one gametocyte was found in one of the slides (at 8 pm on day 2). Morphological staging revealed different proportions of tiny, small and large rings, and mature trophozoites (Figure 1). No schizonts were detected. There was a gradual shift into more mature forms over 48 hour periods. This was most apparent on day 4, with only large rings detected at 2 pm; a microscopy negative sample at 8 pm; and only small rings in the following sample the next day at 8 am.

Multiple *msp1 *and *msp2 *alleles were detected in all *P. falciparum *positive samples (schematically presented in Figure 1). A total of nine different alleles, i.e. PCR products of the same size within the respective allelic types, were found in both loci including all 13 samples. The number of alleles detected in the respective samples ranged between 2 and 7 alleles for *msp1 *(median 5, mean 5.0) and 4 and 6 alleles (median 5, mean 4.85) for *msp2*. Only 2 *msp1 *and 1 *msp2 *allele, respectively, were detected in all consecutive samples. The other alleles were present in different patterns during the five day period. Two *msp1 *and one *msp2 *alleles were detected in only one single sample. The exact same profiles were found in four isolates for *msp1*, two for *msp2*. Including both markers, two consecutive sample pairs had the same patterns (samples day 1 at 8 pm and day 2 at 8 am; and day 4 at 2 am and 8 am). A few alleles were absent in samples collected 48 hours apart. When the profiles were compared in paired consecutive samples, between 0–6 *msp1 *(mean 3) and 0–6 *msp2 *(mean 4) alleles, respectively, were different between two samples collected 6 hours apart.

## Discussion

This detailed assessment reveals extensive dynamics of *P. falciparum *infections with regards to parasite densities, maturation stages and genotyping profiles in an asymptomatic and thus partially immune individual in a high endemic area.

The total parasite densities determined by microscopy fluctuated according to a sine wave curve (Figure 1). The variation in consecutive samples, confirms the limitation of single blood samples to determine the parasite load in an individual [3,10]. A detailed microscopic assessment of the maturation stages distinguished different stages over time with a gradual shift into more mature forms over 48 hour periods, corresponding to the length of the erythrocytic life cycle of *P. falciparum*. On one occasion, the mature stages were followed by a negative sample suggesting synchronized sequestration of the whole parasite population during the later part of the 48 hour life cycle.

Genotyping of *msp1 *and *msp2 *resulted in various patterns in the consecutive samples over five days. The appearance and reappearance of individual clones over this short period suggest that the parasites fluctuated below the threshold of detection mainly due to sequestration. Some of the differences may be explained by variations in the PCR assay. To minimize the impact of methodological factors, all samples were processed together in a highly standardized protocol with high sensitivity of detection (1–10 parasites/microliter as well as detection of parasites in microscopy negative samples). The processing of samples was optimized with the initial steps of DNA extraction performed within one hour after sampling. Small inherent limitations of the genotyping technique, including competition in the PCR amplification due to different proportions of parasite genotypes [11], may still result in small variations in sensitivity resulting in different profiles in consecutive samples.

There was no apparent correlation between the proportion of different maturation forms and the genotyping profiles. The 48 hour patterns found in the previous studies, with daily sampling, suggested that all parasites of a certain clone were sequestered at the same time reflecting synchronization to the same stage of maturation [2-4]. Here, a few clones were absent only in single samples at 48 hour intervals. However, the overall pattern was not as distinct as these previous daily assessments and it was not possible to disentangle whether some clones were more synchronized than others in this short follow up.

In total nine clones, determined by *msp1 *and *msp2 *typing, respectively, were distinguished in this one individual over five days. However, only 2–7 of these alleles were detected in single samples. Determination of the parasite population infecting an individual in single samples, will thus largely underestimate the true number of clones present in an individual at a certain time point. Moreover, the profiles differed by up to six alleles in consecutive samples collected only six hours apart, reflecting that the types of parasites detected in the peripheral blood can differ considerably with only a few hours intervals.

These extensive within-host dynamics of parasite populations are important to consider when interpreting results from genotyping analyses. In drug trials, where genotyping is used to differentiate between recrudescence and reinfection, changes in parasite populations may influence the interpretation of results especially in asymptomatic infections at follow-up [12]. The dynamics of *P. falciparum *infections may vary depending on transmission intensity, immunity, clinical status, as well as methodology [13-17]. With the use of a capillary electrophoresis-based genotyping method, different genotyping profiles were detected over a few hours both in asymptomatic individuals [18] and in symptomatic patients during treatment [19]. Using the current gel-based method, the genotyping profiles were more homogenous in symptomatic infections [16,17]. Here, highly dynamic genotyping profiles was found in an asymptomatic individual in consecutive samples over just a few hours with one of the most widely used methods for genotyping *P. falciparum *infections.

## Conclusion

Although this report describes a *P. falciparum *infection in only one asymptomatic individual over a few days, it highlights the extensive dynamics of parasite populations as well as limitations of single blood samples for establishing parasite densities, maturation stages and genotyping profiles. The findings have implications on a wide range of malaria research studies, and emphasize the importance of detailed assessments of host-parasite interactions. Malaria infections, and what is detected in different assays, may indeed change only over a few hours.

## List of abbreviations

EDTA: ethylenediamine-tetraacetic acid; *msp1*: merozoite surface protein 1 gene; *msp2*: merozoite surface protein 2 gene; PCR: polymerase chain reaction; SDS: sodium duodecyl sulfate; UV: ultra violet light.

## Competing interests

The authors declare that they have no competing interests.

## Authors' contributions

AF planned and conducted the study, and wrote the paper. ML conducted the study and contributed to the preparation of the manuscript. LF planned and conducted the study. IR planned and conducted the study, and wrote the paper. IR was overall responsible for the malaria research projects in Nyamisati.
